# Supplementary material for: Accounting for assay performance when estimating the temporal dynamics in SARS-CoV-2 seroprevalence in the U.S
Source: Nat Commun. 2023 Apr 19;14:2235. doi: 10.1038/s41467-023-37944-5 (PMC10115837; doi:10.1038/s41467-023-37944-5)
Supplement: Supplementary file 2 — Peer Review File [file 41467_2023_37944_MOESM2_ESM.pdf]

nature portfolio

Peer Review File

Accounting for assay performance when estimating the temporal dynamics in SARS-CoV-2 seroprevalence in the U.S

Open Access This file is licensed under a Creative Commons Attribution 4.0 International License, which permits use, sharing, adaptation, distribution and reproduction in any medium or format, as long as you give appropriate credit to the original author(s) and the source, provide a link to the Creative Commons license, and indicate if changes were made. In the cases where the authors are anonymous, such as is the case for the reports of anonymous peer reviewers, author attribution should be to 'Anonymous Referee' followed by a clear attribution to the source work. The images or other third party material in this file are included in the article's Creative Commons license, unless indicated otherwise in a credit line to the material. If material is not included in the article's Creative Commons license and your intended use is not permitted by statutory regulation or exceeds the permitted use, you will need to obtain permission directly from the copyright holder. To view a copy of this license, visit http://creativecommons.org/licenses/by/4.0/.

REVIEWER COMMENTS

Reviewer #1 (Remarks to the Author):

This manuscript describes the importance of serosurveillance for estimating the proportion of the population exposed to SARS-CoV-2 and the limitations and biases in approach to estimate cumulative infections. The authors describe and explain observed geographic and temporal patterns of seroprevalence in the US using CDC's nationwide commercial lab serosurvey data and the impact of differing rates of waning and thus duration of detectability of Ab after exposure in the three assays used. Rates of waning for the three assays were modeled and applied to adjust seroprevalence to estimate the proportion of the population infected. Further, the authors incorporated geographic coverage of vaccination into the proportion exposed over time. This is an important manuscript to examine comparability of population exposure estimates and the impact of different approaches, as it is challenging to reconcile differing estimates reported by multiple studies in the literature understand and respond to the evolving pandemic. The manuscript is generally presented very well, with thorough description of methodology and results, and adequate statement of limitations. The extensive supplemental material is appreciated and useful, particularly where others may want to apply the methodology to other data sets.

The manuscript is appropriate for publication with some suggested clarifications and/or revisions for consideration.

Much of the discussion introduces or reiterates results, new results should be removed from the discussion and reiterating previously described results should be limited to concise summary of results. There is a fair amount of redundancy within and between results and discussion sections. Consider revisions reducing redundancy for conciseness.

Case rates rely on availability of testing, and reporting of cases which may not be consistent over the period of study. How does the increasing use of self testing and likely lower case reporting impact corrections to seroprevalence estimates? Similarly, more specific consideration of the impact of vaccination on rates of waning should be discussed.

Line 30; Please clarify "existing serum and hospital-based samples"

There are significant differences in assay performance related to assay configuration as mentioned by the authors, however the notable similarities to recent infection and differences in waning described (lines 33-44) should clarify that not only does assay format impact these factors (line 35) but importantly Ig type is a main driver of durability of reactivity to S or NC. Thus total (or pan) immunoglobulin assays have significantly longer detection than do IgG assays and thus are more suitable for serosurveillance and extend the period of detectability with minimal waning of reactivity over longer periods, presumably due to maturation of Ab affinity. The point is made somewhat by the authors but it should be clarified that total Ig vs IgG assays perform differently and total Ig assays are more suitable for serosurveillance for this reason.

Line 74; syntax is incorrect in "higher proportions of the Abbott assay were associated with lower seroprevalence while Roche assay use was associated with higher seroprevalence".

Line 242, "It is also important to note that the serosurveys aim to determine evidence of prior infection by detecting the presence of IgG in samples, and as a result, our estimates of the proportion infected, too, focus on estimating prior infections." It is unclear what this statement is trying to convey and it should be corrected as only the Abbott assay detects just IgG.

Line 168; "…we show that estimated proportions infected differ quantitatively and qualitatively from seroprevalence in states that made substantial use of the Abbott assay". Please explain what is meant by a qualitative difference.

Line 214; clarify which independent dataset

Line 246; stating that probabilities of vaccination and infection are independent is somewhat in

conflict with other statements in the manuscript.

Line 254; It is not clear how variation in reporting delays might be a function of the number of cases being reported.

Figure 4 legend refers to the proportion of the population with a complete series of vaccinations, the source of this data should be clarified as elsewhere vaccination status refers to at least one dose.

Please clarify how the blood donor seroprevalence which is reported per study region is compared by state to the lab based study.

The final statement in the discussion mentions that it will become increasingly challenging to understanding infection rates as measured serologically as seropositivity saturates in the population, although the analysis relies on data collected up to January 2022, nearly one year ago, it should be further discussed how variant infection waves may impact rates of waning and how reinfections will be increasingly important but also increasingly difficult to detect.

Reviewer #2 (Remarks to the Author):

Overall, the manuscript reads very well. It’s clearly and carefully written.

I congratulate the Authors for their extensive statistical analysis: retrieving the data, working out the variables, selecting the final models and preparing the very pleasant and pertaining graphical summaries must have taken a considerable amount of time.

To the best of my knowledge, the data used seem appropriate and the methodology is appropriate. I see no major flaw which might invalidate the conclusions.

There are, however, some aspects which are not entirely clear to me and which I would like the Authors to comment upon. Please, refer to the attached referee report.

Reviewer #2 (Remarks to the Author):

Referee Report for Submission NCOMMS-22-40235

Accounting for assay performance when estimating the temporal dynamics in SARS-CoV-2 seroprevalence in the U.S.

Premise

I'm a trained statistician, with working experience in cancer and environmental epidemiology (though limited as far as Covid-19 aspects goes). I restricted myself to the methodological and computational aspects of the statistical analyses, as I don't have the required expertise to evaluate the suitability of the modeling choices nor the relevance of the final results.

Aim and contents

The aim of the paper is to explain the spatio-temporal variation among US States in SARS-CoV-2 seroprevalence, as observed in nationwide sero-surveys conducted for the US CDC in the period July 2020 – January 2022 with three different assays (Abbott, Ortho and Roche). Two logistic regression models ("binomial GLM" in the paper) are selected using three different model metrics (AIC, RMSE and LOO median RMSE): the first includes information on assay use plus further epidemic-related covariates (prevalence of cases, deaths, excess deaths, hospitalization, tests and vaccination plus age distribution of cases); the second tries and models the role played by waning of the antibodies. These models are used to predict the proportions of infected, which are then related to vaccine coverages. The proportions of the populations who received a vaccine prior to infection is furthermore estimated.

Major comments

Overall, the manuscript reads very well. It's clearly and carefully written. (I found only 3 typos, two of which are most likely due to the postprocessing of the original manuscript on the editorial platform). I congratulate the Authors for their extensive statistical analysis: retrieving the data, working out the variables, selecting the final models and preparing the very pleasant and pertaining graphical summaries must have taken a considerable amount of time. To the best of my knowledge, the data used seem appropriate and the methodology is appropriate. I see no major flaw which might invalidate the conclusions.

There are, however, some issues which are not entirely clear to me and which I would like the Authors to comment upon.

1. The models include two types of variables: assay use, which is the "factor" of interest, and the covid-related covariates, which act as "confounders".
   a) What motivated you to use the specific variables listed in the manuscript? Is this topic-related? (If so, please, give some background information for the lay reader like me.) Or, was it dictated by convenience/need (as e.g. these variable are easily accessible)?
   b) Is there a reason why you didn't include into the models some measure of the sensibility/specificity of the three different assays? I understand that this is an ecological study (you use aggregated variables) so the classical formulae which link

these measures to further quantities of interest (prevalence of the disease, predicted values, etc.) are not valid. Nonetheless, I miss the inclusion of some measure of validity of the assays among the covariates of the model.

c) How robust is your model with respect to variable selection? Would your modeling strategy also apply to a different country/countries (provided the corresponding data sources are available)?

2. As far as I understand, the purpose of your models is *prediction*, which places them halfway between a purely descriptive model and an interpretative model. This justifies the use of nonlinear transformations (square root, log) of the original covariates to obtain a better fit. (I appreciate that no polynomials were involved.) But, it entails a number of drawbacks.
   a) We lose the interpretation of both, the variables (what does a unit increase in a square root or a logarithm of a proportion represent?) and of the associated regression coefficients (OR's should be expressed in terms of the original factors). Given the implications of the analysis, the models used should also aim for interpretation and not only be purely descriptive.
   b) Nonlinear transformations are required whenever, as you correctly notice in the paper, relationships are nonlinear. An alternative would be to categorize the continuous covariates, as commonly done in epidemiology. The third option is the one you mention in by-passing, upon which, however, I would base the analysis: GAMs – fitted, however, to the original covariates so as to let the model reproduce the nonlinear relationships.

3. I don't fully capture how you use weighting [lines 350 – 353]:

   "We weighted the model to account for the different proportions of the state populations that were tested in the nationwide serosurveys by scaling the positives and negatives such that the probability that an individual was tested was the same across all states (by using the state population divided by the mean state population as weights in the model)."

   Now, the R help pages report: "For a binomial GLM prior weights are used to **give the number of trials when the response is the proportion of successes**: they would rarely be used for a Poisson GLM." The weights argument may also be used to treat imbalanced data, that is, samples where one of the two outcomes is highly underrepresented. (We may then sample from the outcomes which are more present, fit the model and use the "weights" argument to account for the original frequencies.) Your purpose, however, is to account for the different sampling proportions among the States.
   a) Shouldn't an **offset** be used in this case?
   b) I tried to work out the maths, but didn't succeed. If I understand it right, your models fit P(Test = "positive" | Tested = "yes", State = "i") and are used to predict P(Infected = "true" | State = "i"). The sampling proportions can be written as P(Tested = "yes" | State = "i"). How does the use of the "weights" argumento allow you to link these quantities? And how does the quantity "state population/mean state population" represent/correct for the sampling proportion?

4. What do you mean by "uncertainty interval"? [lines 383 – 387]

"To characterize the uncertainty, we took the best (bottom) five percentile LOO median RMSEs across parameter combinations (times to seroreversion and lead or lag), estimated the proportion infected for the corresponding subset of models to include the 95% uncertainty intervals (UIs) around each model fit, and extracted the range of estimates for each point in time and state (including the 95% UIs)."

a) Are these confidence intervals? If so, provided a proof on their coverage. If not (as I understand it), please, make this clear.
b) Why didn't you use resampling techniques (eg. parametric bootstrap) to get proper CIs?

5. I don't understand what you are aiming for in lines 291 – 296.

"After aggregating the numbers of cases and deaths per state, and differencing the cumulative curves to obtain numbers of cases and deaths per day, we found negative values of both reported deaths and cases. If the negative value was immediately followed by the same (positive) value, those counts were canceled out. Otherwise, the negative total was discounted from previous days' totals. We then aggregated numbers by week, recalculated cumulative numbers, and divided them by the respective state populations to produce cumulative percentages of the population that were reported as COVID-19 cases and deaths, for each nationwide serosurvey round."

6. You made your data available. Does this also hold for the R code? In which form (script, markdown, …)?

<u>Minor issues:</u>

7. The abstract is misleading. It mentions "mechanistic" models, that is, models which try and reproduce the causality of relationships (such as SIR-type models). However, your models are based on associations among the variables and focus exclusively on prediction (not interpretation). Please, fix this.
8. Is there a major reason why you call it "binomial GLM" instead of logistic regression? The latter term is far better known, and more specific.
9. The statement *"the number of positive and negative tests were the response variable"* [lines 340 – 341] is not correct: this simply reflects how the data have to be input to R. The response variable is the number of "successes" (positive tests in this case) out of the total number of tested (which indexes the binomial distribution).
10. You measure the degree of dependence of the covariates using Pearson's correlation coefficient. However, this only holds true if the relationship is linear. Is this the case? Otherwise, use a different measure, such as Spearman's correlation. Furthermore, are all correlations shown in Figure 14 statistically significant?
11. Supplementary Table 1:
    a) *"Exponentiated regression coefficients (risk ratio)"* – What do you mean by risk ratio? Is the rare disease assumption verified (which justifies the interpretation of ORs in terms of RRs)?
    b) Why are the estimates associated with the first five variables (especially for the % of deaths) so different between the reference model and the best waning model?

c) Supplementary Figg. 4 – 6: use the same scale (-0.1 – 0.2) for the third column. Does this column contain the residuals of the model? If so, there seems to be a trend, which is particularly visible in Figure 6. Is this truly the case?
d) Supplementary Fig. 7: What does the blue color represent in the top rows?
e) Supplementary Fig. 10: *"expressed as the interquartile range (IQR) normalized by the median"*. This sounds like a "robust" coefficient of variation. Why not using the common CV (as you used Pearson's correlation coefficient)?
f) Supplementary Fig. 12: What type of smoother did you use? The blue relationship (in the right panel) looks nonlinear, though I believe this may be an artifact due to few outlying and influential observation in the right (which act as leverages). I suggest, if not already done, to use a robust smoother.

Reviewer #3 (Remarks to the Author):

I enjoyed reading this well written, justified, and presented manuscript, and consequently have few comments. I would appreciate it if the authors are able to clarify some points I found unclear.

1. In Fig 3 it appears that the estimated cumulative proportion infected can go down (looking at NE, for example). Can the authors explain why this occurs? Is the model not constrained to only allow this to increase over time?
2. It doesn't appear that the data were sufficient to say anything about multiple infections; can the authors comment about how multiple infections would affect the estimated quantities, such as EPIV?
3. It would be nice if the 3 scenarios presented in Fig 5 were more accurately described in the text; the blue and green ones seem to correspond to the equations on line 396 but the red one is missing. It would improve comprehension to point readers to these equations from the figure caption.

Response to reviewers

We would like to thank all three reviewers for taking time to read through our manuscript; their reviews were thoughtful and very insightful. They raised very good points, and in addressing them, we hope our manuscript is substantially improved. Below, we address each of the points raised by the reviewers in blue text.

Reviewer 1

This manuscript describes the importance of serosurveillance for estimating the proportion of the population exposed to SARS-CoV-2 and the limitations and biases in approach to estimate cumulative infections. The authors describe and explain observed geographic and temporal patterns of seroprevalence in the US using CDC's nationwide commercial lab serosurvey data and the impact of differing rates of waning and thus duration of detectability of Ab after exposure in the three assays used. Rates of waning for the three assays were modeled and applied to adjust seroprevalence to estimate the proportion of the population infected. Further, the authors incorporated geographic coverage of vaccination into the proportion exposed over time. This is an important manuscript to examine comparability of population exposure estimates and the impact of different approaches, as it is challenging to reconcile differing estimates reported by multiple studies in the literature understand and respond to the evolving pandemic. The manuscript is generally presented very well, with thorough description of methodology and results, and adequate statement of limitations. The extensive supplemental material is appreciated and useful, particularly where others may want to apply the methodology to other data sets.

The manuscript is appropriate for publication with some suggested clarifications and/or revisions for consideration.

Much of the discussion introduces or reiterates results, new results should be removed from the discussion and reiterating previously described results should be limited to concise summary of results. There is a fair amount of redundancy within and between results and discussion sections. Consider revisions reducing redundancy for conciseness.

We have reduced the first paragraph of the Discussion, which dealt with sum-

marising the results, to just over half its original length. We have also moved the paragraph on the comparison between New York and New Jersey to the pertinent Results section. Hopefully, these changes will make the Discussion read less like a reiteration of results, and will lessen the feeling of redundancy across sections.

There is one other point in the Discussion where we introduce numbers not previously given in the Results section; these are not new results per se, but rather specific examples from the results that help us make a point.

Case rates rely on availability of testing, and reporting of cases which may not be consistent over the period of study. How does the increasing use of self testing and likely lower case reporting impact corrections to seroprevalence estimates? Similarly, more specific consideration of the impact of vaccination on rates of waning should be discussed.

We agree that given how the pandemic has progressed, more explicit discussion on these points is warranted. We have added a paragraph in the Discussion acknowledging how both reinfections and at-home testing could potentially introduce biases in our estimates of both waning rates and numbers of infections, particularly were our approach to be applied to time periods that extend much beyond January 2022 (at least in the US).

On the second point, we do specifically mention in the Discussion that "Rates of seroconversion and reversion might also be different pre- and post-vaccination", referencing three studies on the subject.

Line 30; Please clarify "existing serum and hospital-based samples"

The surveys used serum samples collected in the provision of healthcare for testing unrelated to SARS-CoV-2; samples were not collected specifically for SARS-CoV-2 surveys. We have edited the text to make it clearer, by referring to

> *"Convenience samples, samples collected from individuals in the provision of healthcare for testing unrelated to SARS-CoV-2,. . . ".*

There are significant differences in assay performance related to assay configuration as mentioned by the authors, however the notable similarities to recent infection and differences in waning described (lines 33-44) should clarify that not only does assay format impact these factors (line 35) but importantly Ig type is a main driver of durability of reactivity to S or NC. Thus total (or pan) immunoglobulin assays have significantly longer detection than do IgG assays and thus are more suitable for serosurveillance and extend the period of detectability with minimal waning of reactivity over longer periods, presumably due to maturation of Ab affinity. The point is made somewhat by the authors but it should be clarified that total Ig vs IgG assays perform differently and total Ig assays are more suitable for serosurveillance for this reason.

We agree that this should perhaps be raised more explicitly. We have added the

following sentence in the Introduction: "That the Abbott assay exhibited faster waning may also imply that the assay immunoglobulin type (IgG in the Abbott, pan-Ig in the Roche) is also important".

Line 74; syntax is incorrect in "higher proportions of the Abbott assay were associated with lower seroprevalence while Roche assay use was associated with higher seroprevalence".

We are unsure where the issue is here. We have tweaked the sentence, changing "Roche assay use" with "use of the Roche assay" and "use of the Abbot assay" in case this was the source of the problem.

Line 242, "It is also important to note that the serosurveys aim to determine evidence of prior infection by detecting the presence of IgG in samples, and as a result, our estimates of the proportion infected, too, focus on estimating prior infections." It is unclear what this statement is trying to convey and it should be corrected as only the Abbott assay detects just IgG.

The meaning was conveyed by the sentence that followed this statement. We wanted to clarify that proportions of populations having been previously infected should not be conflated with proportions of populations with immune protection. Nevertheless, because the focus of the manuscript is on estimating cumulative incidence, we have removed these two sentences to avoid any confusion.

Line 168; "...we show that estimated proportions infected differ quantitatively and qualitatively from seroprevalence in states that made substantial use of the Abbott assay". Please explain what is meant by a qualitative difference.

The quoted text has been removed when reducing the redundancy between the Results and Discussion sections in response to a comment above.

Line 214; clarify which independent dataset

We have added "the nationwide blood donor serosurvey" to the text to clarify.

Line 246; stating that probabilities of vaccination and infection are independent is somewhat in conflict with other statements in the manuscript.

It is the naive, null assumption we make (we specify "we assumed", as opposed to simply state). Indeed, that sentence is immediately followed by one saying that our analyses point to a negative correlation between vaccination and the probability of prior infection.

Line 254; It is not clear how variation in reporting delays might be a function of the number of cases being reported.

This part of the statement is not necessary to the text, so we removed it to avoid confusion.

Figure 4 legend refers to the proportion of the population with a complete series of vaccinations, the source of this data should be clarified as elsewhere vaccination status refers to at least one dose.

The source is the same. As described in the "Data" section:

> *"We produced percentages of the populations that had been vaccinated with at least one dose of a vaccine, or with a complete series of the vaccine (individuals with a second dose of a two-dose vaccine or one dose of a single-dose vaccine)".*

Please clarify how the blood donor seroprevalence which is reported per study region is compared by state to the lab based study.

We describe the approach in the "Data" section:

> *"Multiple estimates were provided for different parts of some states; we took the mean seroprevalence weighted by the number of tests to get a single estimate by state. Surveys were not necessarily performed in the same weeks as the nationwide serosurveys. To maximize the data used when comparing the two datasets, if surveys in the two datasets were performed one week before or after the other, the two values were still matched."*

The final statement in the discussion mentions that it will become increasingly challenging to understanding infection rates as measured serologically as seropositivity saturates in the population, although the analysis relies on data collected up to January 2022, nearly one year ago, it should be further discussed how variant infection waves may impact rates of waning and how reinfections will be increasingly important but also increasingly difficult to detect.

We agree with this point, and in response also to the point raised above, and to a similar point raised by Reviewer 3, we have added a paragraph in the Discussion on these questions.

Reviewer 2

Premise

I'm a trained statistician, with working experience in cancer and environmental epidemiology (though limited as far as Covid-19 aspects goes). I restricted myself to the methodological and computational aspects of the statistical analyses, as I don't have the required expertise to evaluate the suitability of the modeling choices nor the relevance of the final results.

Aim and contents

The aim of the paper is to explain the spatio-temporal variation among US States in SARS- CoV-2 seroprevalence, as observed in nationwide sero-surveys conducted for the US CDC in the period July 2020 – January 2022 with three different assays (Abbott, Ortho and Roche). Two logistic regression models ("binomial GLM" in the paper) are selected using three different model metrics (AIC, RMSE and LOO median RMSE): the first includes information on assay use plus further epidemic-related covariates (prevalence of cases, deaths, excess deaths, hospitalization, tests and vaccination plus age distribution of cases); the second tries and models the role played by waning of the antibodies. These models are used to predict the proportions of infected, which are then related to vaccine coverages. The proportions of the populations who received a vaccine prior to infection is furthermore estimated.

Major comments

Overall, the manuscript reads very well. It's clearly and carefully written. (I found only 3 typos, two of which are most likely due to the postprocessing of the original manuscript on the editorial platform). I congratulate the Authors for their extensive statistical analysis: retrieving the data, working out the variables, selecting the final models and preparing the very pleasant and pertaining graphical summaries must have taken a considerable amount of time. To the best of my knowledge, the data used seem appropriate and the methodology is appropriate. I see no major flaw which might invalidate the conclusions.

There are, however, some issues which are not entirely clear to me and which I would like the Authors to comment upon.

1. The models include two types of variables: assay use, which is the "factor" of interest, and the covid-related covariates, which act as "confounders".

   (a) What motivated you to use the specific variables listed in the manuscript? Is this topic- related? (If so, please, give some background information for the lay reader like me.) Or, was it dictated by convenience/need (as e.g. these variable are easily accessible)?

It is true that the main factor of interest was assay use, but in relation to the other variables used. Of a priori primary concern were the cumulative numbers of reported cases and deaths (as they would likely play an important part in explaining patterns in seroprevalence), but we added the other variables as we assumed they might be important to control for, and because they were available for the US. We have added this sentence for clarification in the "Models" section.

(b) Is there a reason why you didn't include into the models some measure of the sensibility/specificity of the three different assays? I understand that this is an ecological study (you use aggregated variables) so the classical formulae which link these measures to further quantities of interest (prevalence of the disease, predicted values, etc.) are not valid. Nonetheless, I miss the inclusion of some measure of validity of the assays among the covariates of the model.

We believe the different sensitivities and specificities are accounted for by the assay variables. If an assay were to be associated with a significantly lower sensitivity, for instance, it would then be associated with lower seroprevalences. We are not sure what the appropriate way of introducing that information would otherwise have been in this kind of model.

(c) How robust is your model with respect to variable selection? Would your modeling strategy also apply to a different country/countries (provided the corresponding data sources are available)?

The modelling strategy should work for a different country, assuming similar data were available. The variables used in our models include those that one would expect, a priori, to be most important (e.g., reported cases and deaths), and the fact that we recover patterns observed in studies on individual-level data is reassuring. The approach, however, would need to address the questions of reinfections and changing testing strategies (increasing use of at-home testing) if applied to stages of the pandemic that extend beyond the time period analysed here.

2. As far as I understand, the purpose of your models is prediction, which places them halfway between a purely descriptive model and an interpretative model. This justifies the use of nonlinear transformations (square root, log) of the original covariates to obtain a better fit. (I appreciate that no polynomials were involved.) But, it entails a number of drawbacks.

   (a) We lose the interpretation of both, the variables (what does a unit increase in a square root or a logarithm of a proportion represent?) and of the associated regression coefficients (OR's should be expressed in

terms of the original factors). Given the implications of the analysis, the models used should also aim for interpretation and not only be purely descriptive.

(b) Nonlinear transformations are required whenever, as you correctly notice in the paper, relationships are nonlinear. An alternative would be to categorize the continuous covariates, as commonly done in epidemiology. The third option is the one you mention in by-passing, upon which, however, I would base the analysis: GAMs – fitted, however, to the original covariates so as to let the model reproduce the nonlinear relationships.

We understand the rationale laid out here, but understand the purpose of our analyses in a subtly different way. Our objectives were:

- *Characterise and explain the spatio-temporal patterns in seroprevalence in the US, with a particular focus on the role played by the different assays used in shaping those patterns.*
- *Having understood the role played by assays, produce corrected estimates of seroprevalence (or proportions infected, in the manuscript).*

We never intended to focus on interpreting model coefficients, and indeed we make no mention of them in the text. We provide a brief comparison with GAMs to verify that there were no additional non-linearities that could lead to a different interpretation of results. We preferred to focus on the GLMs, because while using GAMs entail the advantages you mention (more naturally dealing with the nonlinear relationships), they come at a cost: with GAMs, we had to make somewhat awkward choices (e.g., keeping a linear interaction term, constraining the degrees of freedom in the splines, and normalising the weights to a mean of one) that could detract from the analyses. The comparison between GLMs and GAMs of Supplementary Fig. 17 shows that there is little difference between the two.

We feel the same argument on why we fit GLMs on transformed variables applies to GAMs too. These variables are multiplicative in nature and are heavily right-skewed, so their transformation is not exclusively a matter of accounting for nonlinearities.

3. I don't fully capture how you use weighting [lines 350 – 353]:

> *"We weighted the model to account for the different proportions of the state populations that were tested in the nationwide serosurveys by scaling the positives and negatives such that the probability that an individual was tested was the same across all states (by using the state population divided by the mean state population as weights in the model)."*

Now, the R help pages report: "For a binomial GLM prior weights are used to **give the number of trials when the response is the proportion of successes**: they would rarely be used for a Poisson GLM." The weights argument may also be used to treat imbalanced data, that is, samples where one of the two outcomes is highly underrepresented. (We may then sample from the outcomes which are more present, fit the model and use the "weights" argument to account for the original frequencies.) Your purpose, however, is to account for the different sampling proportions among the States.

(a) Shouldn't an **offset** be used in this case?

Admittedly, the R help pages are not exhaustive or always clear. As they do specify, though, the weights give the number of trials ***when the response is the proportion of successes*** *(i.e., when the response is expressed as the ratio of successes over number of trials or tests). Our response variable consists of two vectors with the positives and negatives. In this case, the weights do not work as they would if they encoded number of trials. For example, when comparing the following models:*

```
n    = 100
x    = seq_len(n)
succ = rpois(n = n, lambda = 30)                          # Successes
fail = rpois(n = n, lambda = 60)                          # Failures
w    = sample(x = seq_len(n), size = n, replace = TRUE)  # Weights

m1 = glm(cbind(succ, fail) ~ x, family = "binomial")
m2 = glm(succ / (succ + fail) ~ x, family = "binomial", weights = succ + fail)

m3 = glm(cbind(succ, fail) ~ x, family = "binomial", weights = w)
m4 = glm(cbind(succ, fail) ~ x, family = "binomial", weights = w * 2)

m5 = glm(succ / (succ + fail) ~ x, family = "binomial", weights = w)
m6 = glm(succ / (succ + fail) ~ x, family = "binomial", weights = w * 2)
```

`m1` *and* `m2` *are exactly equivalent, but the log-likelihoods of* `m3` *and* `m4` *(which have the dependent variable encoded as columns for successes and failures) are scaled by a factor of two (as are the weights), while for* `m5` *and* `m6` *(where the dependent variable is the proportion of trials that are successful) they are not. The behaviour of weights in other GLMs is not quite like that of* `m3` *and* `m4`*, because the log-likelihoods would come out exactly the same, but presumably that is only because for non-binomial GLMs, weights are internally normalized.*

(b) I tried to work out the maths, but didn't succeed. If I understand it right, your models fit P(Test = "positive" | Tested = "yes", State = "i") and are used to predict P(Infected = "true" | State = "i"). The sampling proportions can be written as P(Tested = "yes" | State = "i"). How does the use of the "weights" argumento allow you to link these quantities? And how does the quantity "state population/mean

state population" represent/correct for the sampling proportion?

You are right that our description in the text is at odds with what weights we actually used; thank you for raising this issue. We had understood that the total number of samples was already present in the model through the dependent variable, but to account for the different sampling proportions, we still need total numbers of samples in the weights too. We do so now, and are more explicit about our approach in the "Methods" section:

> *"We weighted the model to account for the different proportions of the state populations that were tested in the nationwide serosurveys by scaling the positives and negatives such that the probability that an individual was tested was the same across all states. We did so by letting the product of the sampling proportion (number of samples divided by state population) and weights equal the mean sampling proportion across states and rounds."*

This way, the weights compensate for variation in the sampling proportions.

All results have been updated. The patterns have changed little, although the point estimates have. For example, the best model by AIC had a time to seroreversion for the Roche assay of > 97 *weeks in the previous results; now it is 67 weeks. The reason for this is that there are still a fairly large number of models producing similar AICs (the surface in parts of Fig. 2 is relatively flat), so small changes in the results can lead to jumps in the point estimates. Nonetheless, the main take-home message remains: the time to seroreversion for the Abbott assay is shorter than for the Roche assay, and there is no statistical support for there being any seroreversion in the Roche assay (because some models with 97 weeks of time to seroreversion for the Roche assay are still within the best five percentile models).*

4. What do you mean by "uncertainty interval"? [lines 383 – 387]

> *"To characterize the uncertainty, we took the best (bottom) five percentile LOO median RMSEs across parameter combinations (times to seroreversion and lead or lag), estimated the proportion infected for the corresponding subset of models to include the 95% uncertainty intervals (UIs) around each model fit, and extracted the range of estimates for each point in time and state (including the 95% UIs)."*

   (a) Are these confidence intervals? If so, provided a proof on their coverage. If not (as I understand it), please, make this clear.

These are somewhat ad-hoc estimates of uncertainty, as opposed to confidence intervals (hence we say "To characterize uncertainty"). We wanted to provide a sense for the uncertainty in the estimated proportions infected that results, particularly, from the selection of the times to seroreversion. The surfaces of the model performance metrics can be fairly flat in places (Fig. 2), and we wanted our estimates to reflect this. Our mention of "95% uncertainty intervals" refers to the fact that we include the UIs of each individual GLM (each pixel in Fig. 2), when estimating the described cross-GLM uncertainty in the estimated proportion infected.

(b) Why didn't you use resampling techniques (eg. parametric bootstrap) to get proper CIs?

As mentioned in the response above, our approach attempts to quantify uncertainty across different fixed times to seroreversion and lead/lag times. We believe our approach sufficiently characterises that uncertainty.

5. I don't understand what you are aiming for in lines 291 – 296.

> *"After aggregating the numbers of cases and deaths per state, and differencing the cumulative curves to obtain numbers of cases and deaths per day, we found negative values of both reported deaths and cases. If the negative value was immediately followed by the same (positive) value, those counts were canceled out. Otherwise, the negative total was discounted from previous days' totals. We then aggregated numbers by week, recalculated cumulative numbers, and divided them by the respective state populations to produce cumulative percentages of the population that were reported as COVID-19 cases and deaths, for each nationwide serosurvey round."*

The numbers of reported cases and deaths are provided as cumulative numbers. However, those numbers, in few instances, decline, producing negative numbers of (non-cumulative) cases or deaths on a given day. We therefore address this issue by processing the data as described. We are unsure as to how to make the description clearer.

6. You made your data available. Does this also hold for the R code? In which form (script, markdown, ...)?

We initially had intended to provide the data necessary to reproduce the results. However, in view of your comment, we have decided to make R scripts available on Github, together with some of the intermediate output

produced by the scripts.

Minor issues:

7. The abstract is misleading. It mentions "mechanistic" models, that is, models which try and reproduce the causality of relationships (such as SIR-type models). However, your models are based on associations among the variables and focus exclusively on prediction (not interpretation). Please, fix this.

   We used the word "mechanistic" mostly in reference to how waning in assays is incorporated in our modelling approach. Nonetheless, we have followed your suggestion and removed the word when in reference to our approach.

8. Is there a major reason why you call it "binomial GLM" instead of logistic regression? The latter term is far better known, and more specific.

   There is no major reason. We have changed the text, as per your suggestion.

9. The statement "the number of positive and negative tests were the response variable" [lines 340 – 341] is not correct: this simply reflects how the data have to be input to R. The response variable is the number of "successes" (positive tests in this case) out of the total number of tested (which indexes the binomial distribution).

   We have changed that text to say "the number of positives out of the total number of tests were the response variable".

10. You measure the degree of dependence of the covariates using Pearson's correlation coefficient. However, this only holds true if the relationship is linear. Is this the case? Otherwise, use a different measure, such as Spearman's correlation. Furthermore, are all correlations shown in Figure 14 statistically significant?

    We have changed the correlation matrix to use Spearman's correlation coefficients. The vast majority are indeed significant; non-significant correlations ($P > 0.05$*) are blank, although some small but significant coefficients are so faint they could also pass as being blank and therefore not statistically significant.*

11. Supplementary Table 1:

(a) "Exponentiated regression coefficients (risk ratio)" – What do you mean by risk ratio? Is the rare disease assumption verified (which justifies the interpretation of ORs in terms of RRs)?

As we argue in response to another comment, there is no actual intent to interpret the coefficients in the text, so to avoid misunderstandings, we now present the coefficients without exponentiation.

(b) Why are the estimates associated with the first five variables (especially for the % of deaths) so different between the reference model and the best waning model?

As we discuss in response to a point above, the interpretation of the model coefficients was not an objective of the manuscript, and indeed do not discuss them. Comparing the main reference model to the best waning model is tricky, because the "sqrt % cases" variable is very different between the two; in the main reference model, it is a monotonically increasing function of time, in the best waning model, it is not. Furthermore, as you note, the coefficients change for several of the variables, making interpretation of the changes for any one coefficient difficult.

(c) Supplementary Figg. 4 – 6: use the same scale (-0.1 – 0.2) for the third column. Does this column contain the residuals of the model? Ifso, there seems to be a trend, which is particularly visible in Figure 6. Is this truly the case?

We are unsure as to what trend you may be referring to. They are not the residuals of the model; rather, they compare the estimated proportion infected using the best waning model (which is not, to be clear, the fitted values as such, because our predictions replace the "waned" cases with which the model was fit with the cumulative numbers of cases, as described in the "Models" section), with the CDC seroprevalence estimates. After round 24, the Roche assay was used throughout the US, so that the difference between our estimated infections and the CDC seroprevalence drops substantially (as can also be seen in Fig. 3).

(d) Supplementary Fig. 7: What does the blue color represent in the top rows?

It shows how much higher seroprevalence would have been, had a given assay been used exclusively across the US. To clarify, we add the following in the caption: "In the middle and bottom rows, blues show the extent to which seroprevalence would have been higher, had the Ortho or Roche assay, respectively, been used exclusively".

(e) Supplementary Fig. 10: "expressed as the interquartile range (IQR) normalized by the median". This sounds like a "robust" coefficient of variation. Why not using the common CV (as you used Pearson's correlation coefficient)?

Originally, we used the IQR / median precisely because it is a "robust" coefficient of variation. Nonetheless, we have changed the figure to use the common coefficient of variation. The patterns remain largely unchanged.

(f) Supplementary Fig. 12: What type of smoother did you use? The blue relationship (in the right panel) looks nonlinear, though I believe this may be an artifact due to few outlying and influential observation in the right (which act as leverages). I suggest, if not already done, to use a robust smoother.

We used a standard LOESS smoother, with a span of 0.75. We agree that the nonlinearity is likely driven by the outlier values at high seroprevalence in the blood donors dataset. We tried using other, presumably more robust, approaches to fit a nonlinear function (e.g., using `family = "symmetric"` *in the loess function call in R, or using GAMs). They resulted in similar curves, unless we for instance constrained the degrees of freedom in the GAMs to force the function to be (almost) linear. The point remains that those two or three points are the only ones available at the higher values of seroprevalence, allowing for nonlinearity tends to curve the line, and anything we do to prevent that from being the case will also be somewhat arbitrary. These lines were only ever intended as visual guides to show the broad patterns in the underlying data points; they are not used in any other way, so this issue seems of little consequence. If the curve still feels in some way misleading, we could also remove the lines altogether.*

Reviewer 3

I enjoyed reading this well written, justified, and presented manuscript, and consequently have few comments. I would appreciate it if the authors are able to clarify some points I found unclear.

1. In Fig 3 it appears that the estimated cumulative proportion infected can go down (looking at NE, for example). Can the authors explain why this occurs? Is the model not constrained to only allow this to increase over time?

This is a result of the fact that there are many variables in the model, and depending on what those variables do over time, they can lead to the estimated proportions infected going down. This is only ever marginally the case. The model is not constrained to only allow for this estimated measure to increase over time.

2. It doesn't appear that the data were sufficient to say anything about multiple infections; can the authors comment about how multiple infections would affect the estimated quantities, such as EPIV?

We agree with this point, also raised by Reviewer 1; see our response to their comment.

With regards to the EPIV, depending on the correlation structure assumed between probability of infection and vaccination coverage, numbers were already high across the US in January 2022. As a result, with successive waves, and despite reinfections, this number would eventually saturate at or near 100% and remain there. Our estimates are, after all, estimates of proportions of individuals having had some immune response; they do not imply protection. Therefore, although the EPIV might be 100%, the proportion of the population that are protected against infection or severe disease will be lower and change over time.

3. It would be nice if the 3 scenarios presented in Fig 5 were more accurately described in the text; the blue and green ones seem to correspond to the equations on line 396 but the red one is missing. It would improve comprehension to point readers to these equations from the figure caption.

The red points and line use the same equation as the blue ones, except they also include individuals that had a single dose of the vaccine (but not necessarily the full course), while the blue points and line only include individuals with a full course of the vaccine. We now refer to the equations in the caption, as suggested.

REVIEWER COMMENTS

Reviewer #2 (Remarks to the Author):

I congratulate the Authors on their careful revision of the manuscript.

I am very satisfied with how my concerns were addressed. I just would like to ask for some final clarification on one major aspect – the use of the weights argument of the R function 'glm' – and a couple of minor items. Please, refer to the attached referee report.

Reviewer #2 (Remarks to the Author):

Referee Report for Revised Submission NCOMMS-22-40235A

Accounting for assay performance when estimating the temporal dynamics in SARS-CoV-2 seroprevalence in the U.S.

I congratulate the Authors on their careful revision of the manuscript.

I am very satisfied with how my concerns were addressed. I just would like to ask for some final clarification on one major aspect – the use of the `weights` argument of the R function `glm` – and a couple of minor items. The below numeration refers to the original referee report.

Major issue

3.b) You are right that our description in the text is at odds with what weights we actually used; thank you for raising this issue. We had understood that the total number of samples was already present in the model through the dependent variable, but to account for the different sampling proportions, we still need total numbers of samples in the weights too. We do so now, and are more explicit about our approach in the "Methods" section:

"We weighted the model to account for the different proportions of the state populations that were tested in the nationwide serosurveys by scaling the positives and negatives such that the probability that an individual was tested was the same across all states. We did so by letting the product of the sampling proportion (number of samples divided by state population) and weights equal the mean sampling proportion across states and rounds."

This way, the weights compensate for variation in the sampling proportions.

I looked up the R code you provided on GitHub – much appreciated! – but still don't fully grasp how your weighting works... In short,

- the `04_gam_main_reference_model.R` states:

  ```
  weights = (state_population / n_total) / mean(state_population / n_total)
  ```

  I am not an expert in sample surveys, but my very first thought was towards the use of sampling weights in regression to account for different sampling probabilities. However, at item 2.b) you mention that this is part of the awkward choices you had to make to fit the GAMs, such as "normalizing the weights to a mean of one". (Btw, why did you have to do so?)

- the `01_glm_main_reference_model.R` file, on the other hand, states:

  ```
  weights = mean(n_total / state_population) * state_population / n_total
  ```

  I don't think you meant these weights (used in logistic regression) to be the same than those above (for the GAM model fit), which anyway cannot be as

  ```
  mean(n_total / state_population)   ≠   1 / mean(state_population / n_total)
  ```

Any help in shedding some light on this issue would be highly appreciated. In case, while trying and finding my way, I found the following blog very useful (though it didn't entirely unravel my doubts):
https://www.r-bloggers.com/2015/09/linear-models-with-weighted-observations/

The author distinguishes 3 types of weights:

1. *precision weights*, which model the differential precision with which the outcome variable was measured, as implemented in the `weights` argument of the R functions `lm` and `glm`.
2. *frequency weights,* which represent the number of times a particular observation in an aggregated data set was observed. They can be modelled with the `weights` argument of the R function `lm` and `glm`, leading to correct estimates, but wrong inferences unless the degrees of freedom are suitably adjusted.
3. *sampling weights*, which is – I believe – the type of weights you are interested in as they represent the "inverse of the probability of a particular observation to be selected from the population to the sample". **You cannot model these weights with the `weights` argument of the R functions `lm` and `glm`,** but need to rely on other functions such as e.g. those provided by the `survey` **package** (https://stylizeddata.com/how-to-use-survey-weights-in-r/).

Minor issues

1.b) We believe the different sensitivities and specificities are accounted for by the assay variables. If an assay were to be associated with a significantly lower sensitivity, for instance, it would then be associated with lower seroprevalences. We are not sure what the appropriate way of introducing that information would otherwise have been in this kind of model.

Am I right in interpreting your reply that some of the explanatory variables included into the model may act as *proxies* for assay sensitivity/specificity?

2.b) We understand the rationale laid out here, but understand the purpose of our analyses in a subtly different way. Our objectives were:

- Characterise and explain the spatio-temporal patterns in seroprevalence in the US, with a particular focus on the role played by the different assays used in shaping those patterns.
- Having understood the role played by assays, produce corrected esti- mates of seroprevalence (or proportions infected, in the manuscript).

I now understand that the purpose of the analysis is neither descriptive nor interpretative, but predictive. Indeed, this was mentioned in the original submission. I would stress it further in the final version of the manuscript.

4.a) These are somewhat ad-hoc estimates of uncertainty, as opposed to confidence intervals (hence we say "To characterize uncertainty"). We wanted to provide a sense for the uncertainty in the estimated proportions infected that results, particularly, from the selection of the times to seroreversion. The surfaces of the model performance metrics can be fairly flat in places (Fig. 2), and we wanted our estimates to reflect this. Our mention of "95% uncertainty intervals" refers to the fact that we include the UIs of each individual GLM (each pixel in Fig. 2), when estimating the described cross-GLM uncertainty in the estimated proportion infected.

This is not standard methodology, but a heuristic proposal by yours. Please, be clear on this in the final version of the manuscript.

4.b) As mentioned in the response above, our approach attempts to quantify uncertainty across different fixed times to seroreversion and lead/lag times. We believe our approach sufficiently characterises that uncertainty.

Please, define what you mean by “sufficiently”. If not benchmarked, the interpretation of terms like “good”, “well”, “fine”, “sufficient” etc. is subject-specific.
Furthermore, *sufficiency* is a reserved word in statistics: sufficient statistics summarize the information at hand without information loss on the relevant aspects of the problem. I don’t think this is what you are referring to.

6. We initially had intended to provide the data necessary to reproduce the results. However, in view of your comment, we have decided to make R scripts available on Github, together with some of the intermediate output produced by the scripts.

Great! Much appreciated!

8. There is no major reason. We have changed the text, as per your suggestion.

There’s now a major muddle. Your modeling technique is known as **“logistic regression”**. Please, use this term in place of “*logistic generalized linear model (GLM)*”. There is furthermore no need to mention generalized linear models (GLMs), which lay reader may not know (though they may know of logistic regression).

11.e) Originally, we used the IQR / median precisely because it is a “robust” coefficient of variation. Nonetheless, we have changed the figure to use the common coefficient of variation. The patterns remain largely unchanged.

The original proposal was fine! My question was just about its interpretation. Now that you replaced/supported Pearson’s correlation coefficient by/with Spearman’s correlation coefficient (item 10), I would keep to the original (robust) choice.

Reviewer #3 (Remarks to the Author):

All of my concerns were adequately addressed by the authors and I have no remaining comments.

Response to reviewers

We would like to thank both reviewers for reading through our manuscript again, and Reviewer 2 for further comments. We hope to have resolved any outstanding concerns.

I congratulate the Authors on their careful revision of the manuscript.

I am very satisfied with how my concerns were addressed. I just would like to ask for some final clarification on one major aspect – the use of the weights argument of the R function glm – and a couple of minor items. The below numeration refers to the original referee report.

Major issue

3.b) I looked up the R code you provided on GitHub – much appreciated! – but still don't fully grasp how your weighting works... In short,

- the `04_gam_main_reference_model.R` states:

  `weights = (state_population / n_total) / mean(state_population / n_total)`

  I am not an expert in sample surveys, but my very first thought was towards the use of sampling weights in regression to account for different sampling probabilities. However, at item 2.b) you mention that this is part of the awkward choices you had to make to fit the GAMs, such as "normalizing the weights to a mean of one". (Btw, why did you have to do so?)

- the `01_glm_main_reference_model.R` file, on the other hand, states:

  `weights = mean(n_total / state_population) * state_population / n_total`

  I don't think you meant these weights (used in logistic regression) to be the same than those above (for the GAM model fit), which anyway cannot be as

  `mean(n_total / state_population)` $\neq$ `1 / mean(state_population / n_total)`

Any help in shedding some light on this issue would be highly appreciated.

The two sets of weights (GAM and GLM) were indeed different: the weights used in the GLM (say, w) were normalised by the mean weights in the GAM

(w/mean(w)). For the sake of clarity, let N = total number of samples, and P = state population. The weights in the GLM were

$$w = \text{mean}(N/P)\, P/N,$$

(which, as described in the text, aimed to make the probability that an individual is tested the same across all states and equal to the mean sampling proportion). In the GAM,

$$w = \frac{\text{mean}(N/P)\, P/N}{\text{mean}(\text{mean}(N/P)\, P/N)},$$

$$= \frac{\text{mean}(N/P)\, P/N}{\text{mean}(N/P)\, \text{mean}(P/N)},$$

$$= \frac{P/N}{\text{mean}(P/N)}.$$

The reason to normalise the weights (and why it felt like an 'awkward' choice) is that in GAMs, different absolute magnitudes (but same relative differences) of weights lead to potentially quite different outputs (unlike in GLMs). As far as we understand, changing the absolute magnitudes of the weights change the likelihood, which can then lead to the optimisation used by `gam` to change the extent to which nonlinear functions are penalised. This is hinted at in the documentation for the `gam` function, where, with regards to the `weight` parameter, they suggest that

> "If you want to re-weight the contributions of each datum without changing the overall magnitude of the likelihood, then you should normalize the weights (e.g., '`weights <- weights/mean(weights)`')."

In case, while trying and finding my way, I found the following blog very useful (though it didn't entirely unravel my doubts): `https://www.r-bloggers.com/2015/09/linear-models-with-weighted-observations/`

The author distinguishes 3 types of weights:

1. *precision weights*, which model the differential precision with which the outcome variable was measured, as implemented in the weights argument of the R functions lm and glm.

2. *frequency weights*, which represent the number of times a particular observation in an aggregated data set was observed. They can be modelled with the weights argument of the R function lm and glm, leading to correct estimates, but wrong inferences unless the degrees of freedom are suitably adjusted.

3. *sampling weights*, which is – I believe – the type of weights you are interested in as they represent the "inverse of the probability of a particular

> observation to be selected from the population to the sample". You cannot model these weights with the weights argument of the R functions lm and glm, but need to rely on other functions such as e.g. those provided by the survey package (`https://stylizeddata.com/how-to-use-survey-weights-in-r/`).

Thank you for bringing this to our attention. We agree this is the better approach, and have changed our code and results to use the `survey` package. As suggested, we now use the inverse of the sampling proportion as weights. This change does not affect the parameter estimates or predictions, but does increase standard errors and widen uncertainty envelopes around our reconstructions, although in the latter the differences are subtle.

Our analysis using GAMs was only marginal, but for consistency, we have replaced GAMs with splines within the same framework used for the main analysis. The main point we made with the original GAM analysis was to show that allowing for non-parametric nonlinearities in the relationships between the variables and seroprevalence did not significantly change model fit and predicted values relative to the GLMs, and this remains the case using splines.

Minor issues

1.b) Am I right in interpreting your reply that some of the explanatory variables included into the model may act as proxies for assay sensitivity/specificity?

Yes, albeit with a caveat. In the waning models, we still have variables representing the assays, meaning that assays may still be associated with different average seroprevalences, above and beyond the waning component encoded in the assay times to seroreversion. In the main reference model, the assay variables may capture both the waning component (average seroprevalences driven by the various waning rates), and any other differences there may have been between assays after accounting for waning.

2.b) I now understand that the purpose of the analysis is neither descriptive nor interpretative, but predictive. Indeed, this was mentioned in the original submission. I would stress it further in the final version of the manuscript.

We would be somewhat hesitant to define the purpose of the study as being predictive, because that can create the expectation that we will predict future seroprevalences. Here we are, if anything, reconstructing past seroprevalences. To clarify this, we replaced the word "estimating" both at the start of the Abstract and end of the Introduction to "reconstructing".

4.a) This is not standard methodology, but a heuristic proposal by yours. Please, be clear on this in the final version of the manuscript.

This is correct. We now specify

> "To characterize the uncertainty, we used an ad-hoc approach in which…".

4.b) Please, define what you mean by "sufficiently". If not benchmarked, the interpretation of terms like "good", "well", "fine", "sufficient" etc. is subject-specific. Furthermore, sufficiency is a reserved word in statistics: sufficient statistics summarize the information at hand without information loss on the relevant aspects of the problem. I don't think this is what you are referring to.

That may have not been the ideal way to characterise our approach in our response, although those are not the terms used in the manuscript. Given the way in which we model waning in assays, any approach we use to characterise uncertainty will be ad-hoc. Finding a way to quantify that uncertainty in our reconstructions is important, and the approach we use is one of many that might be applied (to go no further, we use the best five percentile models; this figure is arbitrary). In any case, our methodology is transparently laid out.

8. There's now a major muddle. Your modeling technique is known as "logistic regression". Please, use this term in place of "logistic generalized linear model (GLM)". There is furthermore no need to mention generalized linear models (GLMs), which lay reader may not know (though they may know of logistic regression).

For the sake of clarity, we have followed your suggestion and have replaced any references to GLMs with "logistic regressions".

11.e) The original proposal was fine! My question was just about its interpretation. Now that you replaced/supported Pearson's correlation coefficient by/with Spearman's correlation coefficient (item 10), I would keep to the original (robust) choice.

We have reverted the change, as suggested.

REVIEWERS' COMMENTS

Reviewer #2 (Remarks to the Author):

I am entirely satisfied by how the authors addressed my very last questions.
